# Studying structure and functions of cell membranes by single molecule biophysical techniques

**DOI:** 10.52601/bpr.2021.210018

**Published:** 2021-10-31

**Authors:** Qingrong Zhang, Siying Li, Yu Yang, Yuping Shan, Hongda Wang

**Affiliations:** 1 School of Chemistry and Life Science, Advanced Institute of Materials Science, Changchun University of Technology, Changchun 130012, China; 2 State Key Laboratory of Electroanalytical Chemistry, Changchun Institute of Applied Chemistry, Chinese Academy of Sciences, Changchun 130022, China

**Keywords:** Cell membrane, Single molecule, Atomic force microscopy, Super-resolution fluorescence microscopy, Single molecule force spectroscopy

## Abstract

Cell membranes are complicated multicomponent structures, related to many basic cellular processes, such as substance transporting, energy conversion, signal transduction, mechanosensing, cell adhesion and so on. However, cell membranes have long been difficult to study at a single-molecule level due to their complex and dynamic properties. During the last decades, biophysical imaging techniques, such as atomic force microscopy and super-resolution fluorescent microscopy, have been developed to study biological structures with unprecedented resolution, enabling researchers to analyze the composition and distribution of membrane proteins and monitor their specific functions at single cell/molecule level. In this review, we highlight the structure and functions of cell membranes based on up-to-date biophysical techniques. Additionally, we describe the recent advances in force-based detecting technology, which allow insight into dynamic events and quantitativelymonitoring kinetic parameters for trans-membrane transporting in living cells.

## INTRODUCTION

All cells are surrounded by the cell membrane, which separates a cell from the environment and maintains the fundamental differences between the cytosol and the extracellular environment (Glancy and Balaban 2012; Raposo and Stoorvogel 2013; Simons and Toomre 2000). Membranes serve as many crucial biological functions, such as forming barriers between extracellular and intracellular environments, regulating the transport of substances (Jiang *et al*. 2003), mediating the communications between cells (Lin *et al*. 2004), identifying and transmitting electrical/chemical signals through protein receptors (Rivière *et al*. 2009). Due to its vital role in a variety of cellular activities, the cell membrane is one of the most attractive topics for multidisciplinary studies, including biology and physics. Cell membranes are fluidic and dynamic, and most of their molecules can move in the plane of the membrane (Nicolson 2014). Because of the structure region executing specific functions at the nanoscale and millisecond level in cell membrane, the investigation of membrane structure and functions requires a number of techniques with a high spatiotemporal resolution to directly image cell membranes under near physiological conditions (Sengupta *et al*. 2012). In the past decades, single molecule biophysical techniques offered unparalleled advantages to study the structure and function of cell membranes. Here, we focus on reviewing the researches based on optical-based super resolution fluorescence imaging techniques and force-based microscopies, which can monitor the structure and functions of cell membrane at a single particle/molecule level.

## HISTORY OF THE STRUCTURE OF CELL MEMBRANES

**Figure 1:**
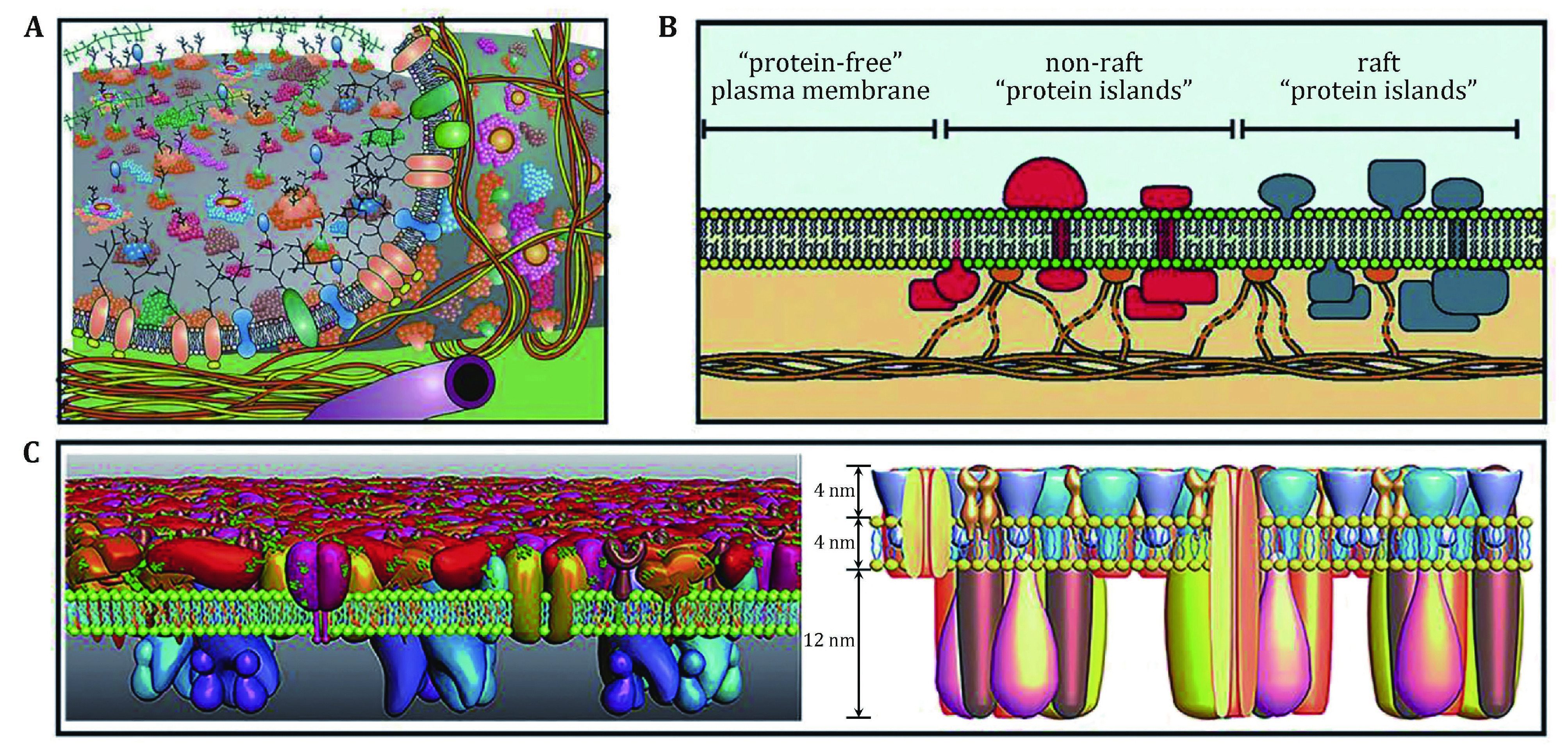
The proposed models of cell membranes. **A** An updated fluid mosaic membrane model that contains information on membrane domain structures, membrane-associated cytoskeletal, and extracellular structures. Reprinted from Nicolson (2014) with permission from Elsevier. **B** Lipid raft domains in cell membranes. Reprinted from Lillemeier *et al*. (2006) with permission from National Academy of Sciences, U.S.A. **C** Proposed protein layer-lipid-protein island (PLLPI) model of the cell membrane. The proteins on the ectoplasmic side of the cell membrane form a dense protein layer to show a smooth feature; the proteins on the cytoplasmic side tend to form dispersed microdomains (left). The total height of the cell membrane is 20 nm, which is composed of ectoplasmic protein layer (4 nm), lipid bilayer (4 nm), and cytoplasmic protein layer (12 nm) (right). Reprinted from Zhao *et* *al*. (2014) with kind permission from the Public Library of Science

Gerle provided a historical outline on biomembrane structure in the last 100 years (Gerle 2019). In 1925, Gorter and Grendel noticed the molecular structure of plasma membranes, and they believed that the cells are surrounded by a lipid bilayer with the hydrophilic components in the external and hydrophobic components in the internal of the membrane (Gorter and Grendel 1925). Danielli and Davson proposed a sandwich membrane model (Davson–Danielli model or the protein–lipid–protein model) in 1935 (Danielli and Davson 1935). Following the development of immuno-electron microscopy (immuno-EM) and freeze-fracture technique, Singer and Nicolson proposed the celebrated fluid mosaic model (FMM) of biological membranes in 1972 (Singer and Nicolson 1972), and the FMM of membrane structure still relevant to understanding the structure, functions and dynamics of biological membranes after more than 40 years (Anderson 2007; Goni 2014; Mouritsen 2011; Nicolson 2014) (Fig. 1A). With the biophysical techniques emerging, such as detergent extraction, centrifugation, and cholesterol depletion by methyl-beta-cyclodextrin (Brown and Rose 1992), Simons and Ikonen postulated particular functional aspects of specialized domains called lipid rafts in biological membranes (Fig. 1B) (Simons and Ikonen 1997). The recent development of super-resolution imaging further confirmed the existence of lipid rafts (Lillemeier *et al*. 2006). Based on the observations of *in situ* AFM imaging and super-resolution fluorescence microscopy (SRFM), Wang *et al*. proposed a semi-mosaic model for red blood cell (Wang *et al*. 2010) and a protein layer–lipid–protein island (PLLPI) model for mammalian nucleated cell membranes (Zhao *et al*. 2014). As shown in Fig. 1C, the PLLPI model highlights that the dense protein layer is the main functional component in terms of mechanical properties, signaling transduction, and substance transporting. The semi-mosaic model and PLLPI model clearly indicated the universality of membrane asymmetry and the diversity of various membranes.

## METHODS FOR STUDYING THE STRUCTURE AND FUNCTIONS OF CELL MEMBRANE

Currently, there are a large number of techniques available for studying cell membranes *in situ.* We mainly summarized imaging techniques here, and X-ray diffraction, IR, Raman spectroscopy, and other biochemical approaches are not reported in this review (Jaumot *et al*. 2004). Some biochemical treatments such as sodium dodecyl sulfate (SDS) and Triton treatment can influence the structure of cell membranes (Bustin 2015). Conventional fluorescence imaging techniques show the capability of directly imaging membranes *in vivo*; however, they suffered from diffraction limit (Abbe 1873) and failed to resolve domains of nanometric size. Time consuming and destructive sample preparation can occasionally lead to low specificity and errors in electron microscopy detecting (Cheville and Stasko 2014). Here, we summarize the exciting single molecule biophysical approaches, which are used to the morphological mapping of a wide variety of proteins and carbohydrates on cell membranes, and the characterization of the functional response of cell membranes under physiological conditions.

### Optical-based microscopy

To study the cell membrane without being invasive, ﬂuorescence microscopy is the ideal tool. However, it is difficult to obtain the structure of all molecules on the cell membrane by a conventional optical microscope at nanometer precision due to the optical diffraction limit (Abbe 1873). Thus, distinct methods with higher resolution and suitable sample preparation are required to directly visualize and pick up fine details of the proteins and carbohydrates on cell membranes (Mateos-Gil *et al*. 2016). Besides, small molecule fluorescence probes and the emit light at the near infrared range also make exploring the proteins and carbohydrates on cell membranes possibly (Thomas 2015).

The super-resolution fluorescence imaging techniques have broken through the optical diffraction limit and greatly improved the spatial resolution to tens of nanometers during the past decades (Huang *et al*.^2010^). These methods provide a powerful tool for studying the distributions and functions of proteins and carbohydrates on cell membranes (Jacobson *et al*. 2019). For example, the synaptosome-associated protein receptor syntaxin-1A (van den Bogaart *et al*. 2011) and glycans (Letschert *et al*. 2014) are distributing in clusters on the plasma membrane demonstrated using super-resolution fluorescence microscopy.

One branch of efforts focused on the diffraction-limited “far-field”, in which super resolution microscopy can probe the localization of single molecules with a resolution in the range of a few tens of nanometers (Huang *et al*. 2009). Among “far-field” microscopy, one commonly used optical microscopy is single molecule localization-based microscopy (SMLM) such as stochastic optical reconstruction microscopy (STORM), photoactivated localization microscopy (PALM) and other derivatives (Betzig *et al*. 2006; Heilemann *et* *al*. 2008; Hess *et al*. 2006; Rust *et al*. 2006). STORM and PALM obtain the stochastical location of molecules by turning on individual molecules within the diffraction-limited volume at different time points and then reconstruct the stochastical location of molecules to achieve high resolution images (Huang *et* *al*. 2010; Sahl *et al*. 2017). When isolated in space, the positions of individual molecules can be determined at nanometer or even sub-nanometer precision by localizing the center positions of their images (Moerner and Orrit 1999; Pertsinidis *et al*. 2010; Yildiz *et al*. 2003). Molecules within the same diffraction-limited volume generate overlapping images, which is the fundamental cause of the diffraction limit in resolution. STORM and PALM overcome this limit by switching on only a stochastic subset of fluorescent molecules within a field of view at any given time, such that their images do not substantially overlap, allowing their positions to be localized with high precision (10–20 nm resolution) (Patterson *et al*. 2010). These molecules are then switched off (or bleached) and a stochastically different subset of molecules is switched on, localized-iterating this process allows a super-resolution image to be constructed from numerous molecular localizations accumulated over time (Betzig *et al*. 2006; Hess *et al*. 2006; Rust *et al*. 2006). Such stochastic activation of molecules is typically achieved by using photo-switchable dyes or fluorescent proteins (Patterson *et al*. 2010). A variety of photo-switchable probes have been used for this approach, in some cases leading to the creation of different names subsequently, but the imaging principle is the same as that of STORM and PALM (Sharonov and Hochstrasser 2006).

As another category of methods in the “far-field” microscopy, stimulated emission depletion (STED) microscopy overcomes the diffraction barrier by optically confining the excitation beam in confocal microscopy to a spot smaller than a diffraction-limited area (Hell and Wichmann^1994^). As a result, only molecules at the very center of the donut shaped beam (where the laser intensity is near zero) can emit light, thus creating a region of fluorescence emission that is much smaller than a typical focal spot of the optical microscope. The reverse strategy is also possible, with the donut beam serving as patterned activation rather than depletion, limiting the emission-free region instead of emission region to the center of the beam (Eggeling *et al*. 2015), scanning these beams across the sample then generating an image with a much higher resolution than the diffraction limit (Hell and Wichmann 1994).

Various other illumination patterns can also be used to increase the spatial frequency of the emission region and hence the image resolution. For example, in structured illumination microscopy (SIM), the sample is excited by a series of standing waves with different orientations or phases to increase the spatial frequency detectable by the microscopy (Gustafsson 2000; Heintzmann and Gustafsson 2009; Schermelleh *et al*. 2008). Because the standing-wave pattern is itself limited by diffraction, the linear form of SIM only extends the diffraction limit, whereas the nonlinear form of SIM (NL-SIM) overcomes the diffraction limit by using the nonlinear or saturated response of fluorophores to further increase the spatial frequency of the emission pattern (Heintzmann and Gustafsson 2009). Unlike STED, which generates super-resolution images directly from the recorded raw data, SIM and NL-SIM require additional computational treatment to reconstruct final images (Rego *et al*. 2012).

In addition, Förster resonance energy transfer (FRET) is a key tool for investigating membrane structure and composition (Saha *et al*. 2015; Sharma *et al*. 2004). The spatial regime probed by this technique makes it ideal for studying nanoscopic domains, and it has been applied to study the model of membranes (Pathak and London 2015) and live cells (Engel *et al*. 2010), not only to study the existence of domains but also define their size (Heberle *et al*. 2010; Pathak and London 2015) by using the fluorescent probes with different FRET efficiencies. FRET is an established photophysical phenomenon to monitor molecular dynamics and interactions at the single molecule level (Deindl and Zhuang 2012). The mechanism is based on the energy transfer from an excited fluorescent donor molecule to an acceptor molecule in a nonradiative fashion when they are in close proximity (1–10 nm) (Förster 1948).

Another branch of efforts is concentrated on the “near-field”, in which a tiny probe is placed near the sample and scanned, thus providing images of surfaces with a resolution that is no longer limited by diffraction (~20 nm resolution) (Dürig *et al*. 1986). Among “near-field” microscopy techniques, near-field scanning optical microscopy (NSOM) is particularly well suited for studying cell membrane as it can typically reach a higher resolution for surface components of cell membrane. NSOM is microscopy without lens (Edidin 2001), wherein the illuminating light is brought in close proximity (a few nanometers) to a sample surface through an aperture with a diameter far less than the wavelength used. This technique enables the illuminating light to reach the sample surface before it is diffracted or lost in the “far-field” spectrum, thereby generating an image with a resolution much higher than that of traditional optical microscopy (Michaelis *et al*. 2000).

### Force-based microscopy

Unlike optical-based microscopy detection, force-based microscopy detection is not limited by optical diffraction. Several force-based detection methods are summarized below. Among all force-based microscopy, AFM is one of the most ideal techniques (Binnig *et al*. 1986). AFM allows the observation of cell membrane in their native environment at a signal-to-noise ratio superior to that of any optical microscopic technique (Binnig *et al*. 1986). The key breakthrough is the realization that samples can be imaged at atomic or molecular resolution, without using an incident beam of photons or electrons, but by measuring the “near-field” interaction between the AFM tip and the sample surface (Muller and Dufrene 2008). AFM is commonly used for imaging the topography of biological structure, the resolution is hundreds of times better than that allowed by the optical diffraction limit. As the technique shows exquisite sensitivity, it is ideally suited for imaging cell membranes (Alsteens *et al*. 2017a; Dufrene 2008; Engel and Muller 2000; Shan and Wang 2015).

Besides imaging, AFM may also be used as a force detector, directly obtaining information on the localization, adhesion, elasticity, and interaction of molecules (Hinterdorfer and Dufrene 2006; Muller *et al*. 2009). In the force spectroscopy mode, interaction forces are measured by recording the deﬂection of the AFM tip cantilever while the tip moved up and down, thus yielding a force-distance curve (Oesterhelt *et al*. 1999). Furthermore, acquiring force-distance curve makes it possible to map interactions between molecule and molecule/cell (Gaboriaud *et al*. 2008). Mapping and functionally analyzing single molecules using so-called single-molecule force spectroscopy (SMFS) requires modification of the AFM tip with specific ligands, like antibodies or lectins, and then measuring the specific interaction force between the ligand and its receptor (Strunz *et al*. 1999). Functionalization of the AFM tip is achieved using cross-linker molecules that anchor ligands firmly at low density, while maintaining their mobility and functionality (Barattin and Voyer 2008). SMFS can help us understand cell surface proteins such as adhesions assemble into nanodomains on the surface of living cells (Alsteens *et al*. 2010; Dupres *et al*. 2005). SMFS may also be exploited to pull on single molecules in order to study their elasticity, a property that plays an important role in cell behavior (Dupres *et al*. 2009). To understand the dynamic process during cell entry in living cells and avoid disturbing AFM tip cantilever moving, a technique based on SMFS, force tracing, was developed by Wang *et al*. (Pan *et* *al*. 2015). During the force tracing test, the AFM tip cantilever bends downward, which causes a transient deflection; subsequently, the feedback system adjusts the position of the piezoelectric ceramic to maintain the deflection of the cantilever, which could be converted to force (Pan *et al*. 2015). The change of position during substance entry cell can also be monitored using constant force mode. The performing details of the force tracing technique are described as following: (1) Before performing the force tracing test, the AFM tip connected with the target substance is located above the relatively flat region of cell periphery with the help of CCD observation; (2) The target substance modified AFM tip moves toward the cell surface and contacts the living cell at a constant force/position through the fine-tuning of the feedback system, then the feedback system is closed; (3) In constant position mode, the force and duration of substance attached on AFM tip entry into cells will be detected by recording the deflection of the AFM tip cantilever and collecting by a PCI data acquisition card (Pan *et al*. 2017). The force tracing shows unique advantages for detecting the force, duration, and displacement during the dynamic membrane transporting process at single molecules/particles level in living cells.

Optical tweezers or optical traps exploit the fact that light exerts a force on the matter (Zhang and Liu 2008). Dielectric particles, such as uniform beads or cells, are attracted and trapped near the waist of a laser beam that has been focused through a microscope objective. Applied external forces will displace a trapped bead from the trap center, with a linear dependence of displacement on force (Simmons *et al*. 1996). Such traps can be made sufficiently compliant so that they exert little resistance to the movement produced by single molecules. Biological macromolecules can be bound to polystyrene or silica beads, which are usually ~1 µm in diameter (Maimaiti *et al*. 2015). A trap can then be moved to steer a bead into a desired experimental geometry (for example, to interact with a partner molecule attached to a coverslip). Upon binding between the two molecules, the forces and movements involved can be measured, and the interaction can be perturbed by moving the trap (Capitanio and Pavone 2013).

Similarly, magnetic tweezer is also a force-based measurement technique. A magnet is positioned above a flow cell that is placed on an inverted microscope. The magnetic force field is usually generated with a pair of permanent magnets, while implementations based on electromagnets (Fisher *et al*. 2006; Gosse and Croquette 2002; Haber and Wirtz 2000) or the “near-field” of a single permanent magnet (Yan *et al*. 2004) have also been reported. The applied magnetic field induces a magnetic moment in the paramagnetic bead, and the bead experiences a force proportional to the gradient of this field. The force between 10 and 100 pN can be readily exerted on paramagnetic beads with a diameter of 1 to 3 μm (Kollmannsberger and Fabry 2007; Lipfert *et al*. 2009) by using magnets that are conveniently positioned outside of the flow chamber. The characteristic length scale over which the magnetic field gradient varies is large, typically on the order of 1 mm. As a result, the generated force can be at a good approximation assumed constant over the length scale that a tethered bead moves. Magnetic tweezers thus provide an infinite bandwidth force clamp without the necessity of sophisticated active force feedback required with optical tweezers (Neuman and Nagy 2008). Taken together, the optical tweezers and magnetic tweezers are similar to the function of AFM-based SMFS (Neuman and Nagy 2008).

## STUDYING ON THE STRUCTURE AND FUNCTIONS OF CELL MEMBRANES

### The distribution of proteins and carbohydrates on cell membranes

The proteins and carbohydrates are important components of cell membranes and also undertake primary functions for cell membranes. Exploring the composition and distribution of proteins and carbohydrates on cell membranes is essential to explain their functions (Bretscher and Raff 1975).

**Figure 2:**
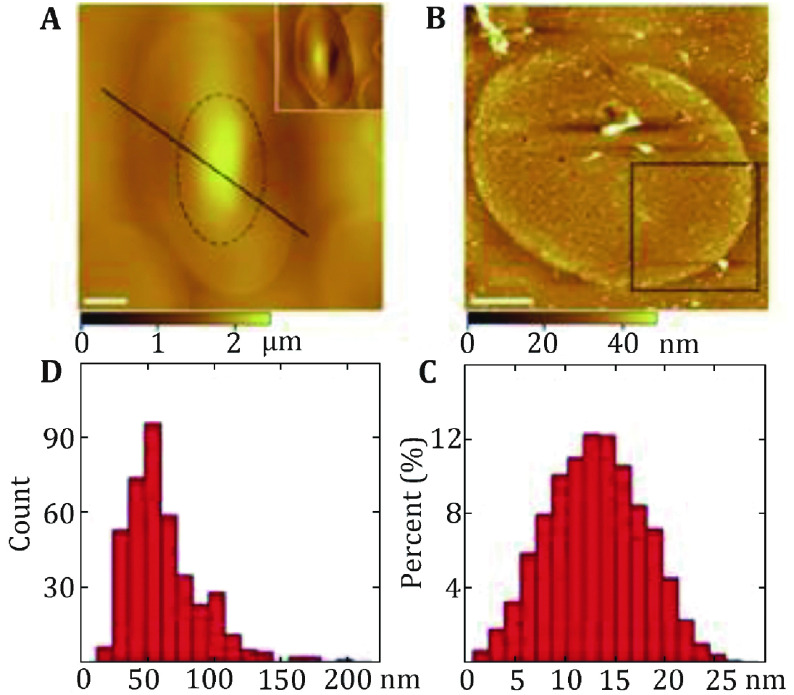
Characterization of the protein-covered cytoplasmic side of the turtle erythrocyte membranes. **A** AFM topographic images of the smooth outer surface of the turtle erythrocytes membranes. **B** AFM topographic image of the cytoplasmic side membrane. Scale bar: 5 µm in **A** and **B**. **C** The height of proteins above the membrane ranges from 1 to 27 nm with the peak at 10–16 nm. **D** The diameter distribution of proteins in the cytoplasmic side membrane varies from 15 to 200 nm with 70% falling between 40 and 80 nm. Reprinted from Tian *et al*. (2014a) with permission of Korean Society for Molecular and Cellular Biology

Cell membranes, which are prepared by the shearing open method (the inner membrane leaflet was prepared by shearing open adsorbed erythrocytes with a fast stream of hypotonic buffer through a needle at a 20-degree angle), have been directly visualized by AFM. Various types of proteins can be found at the cytoplasmic side of the cell membrane, such as the intracellular domains of receptors and transporters. The inner membrane leaflet is covered by dense proteins with fewer free lipids than expected (for example the turtle cell, Fig. 2A). In contrast, the outer membrane leaflet is quite smooth, oligosaccharides and peptides supposed to protrude out of the outer membrane leaflet surface might be actually hidden in the middle of hydrophilic lipid heads (for example the turtle cell, Fig. 2B). Trans-membrane proteins might form domains in the membranes revealed by PNGase F and trypsin digestion on the human red cells (Wang *et al*. 2010). In turtle cells, the proteins in the cytoplasmic side of the cell membrane display a broad range in size and shape, with diameters of 15–200 nm with 70% falling between 40 and 80 nm (Fig. 2C and 2D) (Tian *et al*. 2014a). The height of the protein particles on the membranes varies from 1 to 27 nm with a peak at 10–16 nm. In chicken erythrocytes, the height of the protein particles on the cell membrane varies from 1 to 35 nm with the peak at 10–13 nm. These proteins display a broad distribution of diameter from 45 to 250 nm with 75% in the range of 55 to 100 nm (Tian *et al*. 2014b). The height of the cytoplasmic side of cell membranes between the substrate and the proteins is 18.2 ± 3 nm at erythrocytes from crucian carp. The height of the protein particles on the membrane varies from 1 to 35 nm with the peak at 10 to 13 nm (Tian *et al*. 2014c). These protein particles display a broad distribution of diameter from 30 to 200 nm with 60% in the range of 70–115 nm. Similarly, the average height of the membranes is 19.5 ± 2.8 nm for the nucleated mammalian cell membranes (Zhao *et al*. 2014). The cytoplasmic side of cell membranes is rather rough and covered with proteins, which can be seen more clearly in the magnified image.

To explore the arrangement of proteins and the lipid bilayer of cell membranes at the molecular level, researcher treated the cytoplasmic side of the cell membrane by proteinase K that can digest most of the membrane proteins (Tian *et al*. 2014b, c; Zhao *et al*. 2014). The topography of the digested cytoplasmic side of the cell membrane indicates that many proteins have been removed, and only separated peptides or proteins are visible right above the lipid bilayer. There are some free lipid bilayers at the edge of the membranes due to the removal of membrane proteins. These results provide additional evidence for the arrangement of proteins above the lipid bilayer. The height of the lipid bilayer is 2.5 ± 0.5 nm, which is in complete accordance with a previous value measured with AFM (Wang *et al*. 2010). The height distribution of undigested proteins and peptides above the membrane is distinctly lower than that of the untreated membrane. Recently, the same distribution of protein clusters has also been found on the cytoplasmic side of the mononuclear-macrophage membrane. These protein clusters range in size from tens to hundreds of nanometers. The clustered distribution of proteins in the cell membrane is common, and protein clusters may serve as functional units to participate in various physiological activities.

The ectoplasmic side of erythrocyte membranes is rather smooth, and its average roughness is approximate 0.2 nm, which is much less than that of the cytoplasmic side membrane (Wang *et al*. 2010). The findings suggest that the membrane protein distribution is much more asymmetrical than previously proposed. AFM can also observe any obvious protrusion on the membrane, the blood group B antibody was adsorbed onto the ectoplasmic side membrane of B-type human red blood cells. There is a single antibody particle on the cell membrane, which supports that there is no protein protrusion out of the membranes (Wang *et al*. 2010; Shan *et al*. 2010).

**Figure 3:**
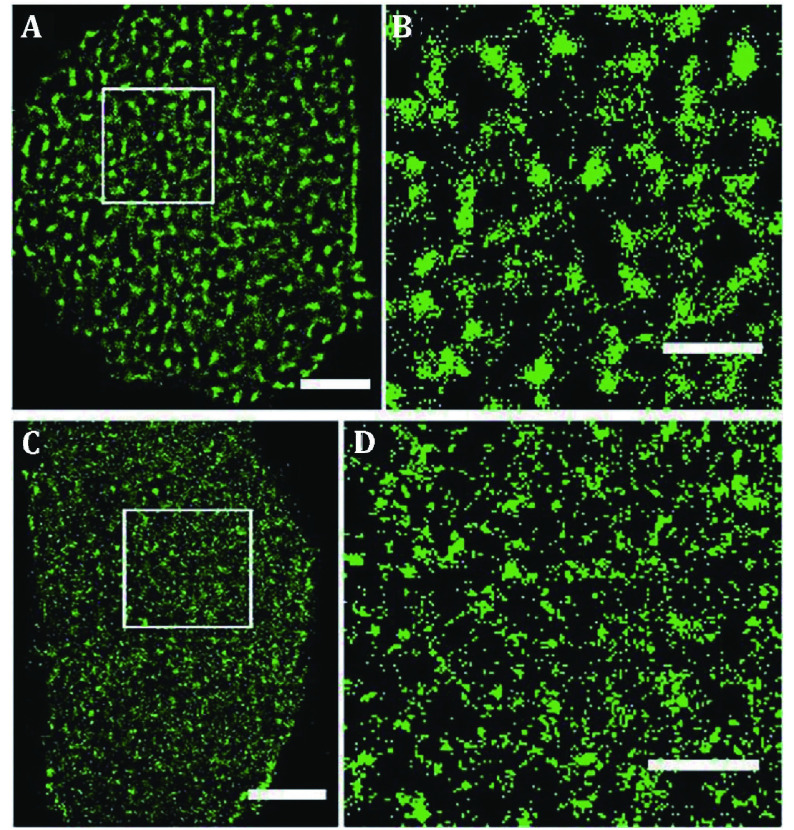
GLUT1 proteins form clusters of different sizes and amounts on HeLa cell medium exposed side and adherent side. GLUT1 antibody was labeled with Alexa532. Reconstructed dSTORM images of GLUT1 on medium exposed side (**A**) and adherent side (**C**). The corresponding magnified images (**B** and **D**). Scale bars: **A** and **C**, 5 μm; **B** and **D**, 2 μm. Reprinted from Yan *et al*. (2018) with permission of National Academy of Sciences, USA

The nanoscale organizations of membrane proteins have further been directly visualized on tissue cells by super-resolution fluorescence microscopy, including glucose transporter 4 (GLUT4) (Gao *et al*. 2017), receptor proteins (Gao *et al*. 2015; Kellner *et al*. 2007; Rogacki *et al*. 2018; Sherman *et al*. 2011), enzymes (Wu *et al*. 2013a), immune proteins (Gomes de Castro *et al*. 2019; Lillemeier *et al*. 2010; Sanchez *et al*. 2019; Scarselli *et al*. 2012), and adhesions (Betzig *et al*. 2006; Shroff *et al*. 2007). All these proteins have been found to gather into clusters on tissue cells. The clustered distribution of proteins in the cell membrane is common, and protein clusters may serve as functional units to participate in various physiological activities (Hess *et al*. 2007). Wang *et al.* reported the clustering property of membrane proteins, such as epidermal growth factor receptor (EGFR) is forming clusters with different sizes and numbers in the distinct membrane background of the polarized cells, which is associated with the external environment, cell conditions, cell types (Gao *et al*. 2015), and ionic protein-lipid interaction (Wang *et al*. 2014). The glucose transporter 1 (GLUT1), transporting the cellular basic energy, is also clustered on cell membranes. Yan *et al.* labeled GLUT1 using GLUT1 antibody and then imaged GLUT1 by dSTORM. Their results showed that GLUT1 tends to form elliptic and dense clusters on the medium exposed side (Fig. 3A and 3B), but sparse clusters with irregular shapes on the adherent side (Fig. 3C and 3D). The same phenomenon was also observed on OS-RC-2 cell (human renal carcinoma cell) membranes (Yan *et al*. 2018). The GLUT1 form clusters with an average diameter of 250 ± 20 nm on the medium exposed side and 137 ± 16 nm on the adherent side, that is, GULT1 form much more and larger clusters on the medium exposed side than that on the adherent side (Fig. 3C and 3D). Further study revealed that not only the “lipid rafts” environment can stabilize their existence, but also the N-glycosylation plays important roles in the clusters formation. These findings have also shown that the size of the protein clusters is in the range of a few hundred nanometers, which is similar to the results obtained from “far-field” optical microscopy. The “near-field” optical microscopy has also provided the evidence that protein aggregated clusters on the cell membrane (van Zanten *et al*. 2009; van Zanten *et al*. 2010).

**Figure 4:**
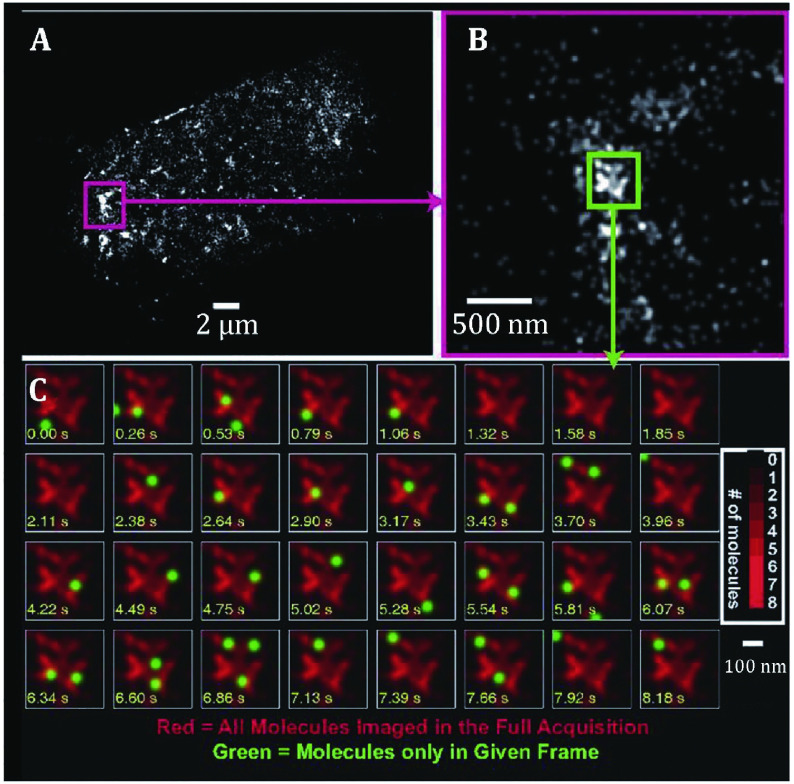
Time dependence of positions of localized HA molecules within an HA cluster in a live fibroblast at room temperature. **A** PALM image of a whole cell. **B** Zoomed view of area in **A** enclosed in dashed magenta box. **C** Successive frames from the 0.4 µm × 0.4 µm region outlined by the green box in **B**. Time is shown on each frame in seconds (yellow text); molecules localized in the current frame are shown as green spots superimposed on a red image of all molecules localized within that region during the full acquisition. Many molecules are visible for more than one frame (typically two to five frames) before photo bleaching. Molecules are plotted as Gaussian spots with a radius of 40 nm. Brighter regions in the image correspond to larger numbers of molecules (see color bar). Reprinted from Hess *et al*. (2007) with permission of National Academy of Sciences, USA

The proteins clustered distribution is slightly different from that of living cells due to cell membrane is dynamically changed. The positions of individual localized photoactivatable green fluorescent protein tagged influenza hemagglutinin (PA-GFP-HA) molecules were plotted as a function of time, along with the overall distribution of molecules ultimately obtained (Fig. 4A and 4B). Molecules are frequently visible for two to four successive frames, during which time (150–600 ms) they move on ~100 nm. However, these motions were found to be spatially constrained and did not span the entire lateral space available within the membrane (Fig. 4C). Instead, motions mapped out regions with elongated shapes and irregular boundaries, suggesting that HAs may move along one-dimensional paths within the two-dimensional membrane. Alternatively, because some membrane regions appear to be inaccessible to HA, mobile HA molecules may undergo tethered diffusion (Hess *et* *al*. 2007). Such results are consistent with observations by confocal microscopy and EM that the clustered distribution of HA leads to a significantly lower density for many areas than the average density. Although individual protein moves in local small regions (Manley *et al*. 2008), the proteins as a whole are distributed in clusters at the plasma membrane of living cells (Gudheti *et al*. 2013; Hein *et al*. 2010; Shroff *et al*. 2008).

Interestingly, carbohydrates have also been found to gather into clusters. Researchers further systemically investigated the spatial organization of the other six representative carbohydrates by dSTORM (Chen *et al*. 2016), and they found that all of the carbohydrates distributed in clusters on the cell membrane and connected with each other to form a conjoined functional platform. The carbohydrate clusters can stably exist with limited size with the contributions of lipid rafts as the stable factor and actin cytoskeleton as the restrictive factor. Recently, Möckl *et al.* have combined bio-orthogonal chemistry with quantitative super-resolution imaging to investigate the nanoscale organization of glycocalyx and its relationship to oncogenic cellular transformation. The findings showed that the density, clustering, and contact sites of glycans and their interaction partners in the context of both health and disease cells are especially attractive as future avenues (Mockl *et al*. 2019).

One of the main purposes to study the protein and carbohydrate clusters are to understand their formation and regulation mechanism (Brewer *et al*. 2002). The size of protein and carbohydrate clusters is an important parameter, which indicates the existing mode and density, and simultaneously also reflects the primary function of cell membranes (Fujita *et al*. 2009). For cell membrane imaging, the AFM imaging could provide higher resolution to visualize the arrangement of proteins and the lipid bilayer of cell membranes at the molecular level, but shows disadvantages in identifying the proteins. However, super-resolution microscopy is good at identifying the proteins by fluorescence labeling, although with a little bit lower imaging resolution than that of AFM. If further scaled up to allow systematic, multiplexed specific imaging, it is possible to investigate the spatial organization of protein and carbohydrate clusters coupled to specific physiological function activities.

### Studying the functions of cell membranes

As a highly heterogeneous and dynamically organized entity in the cells, the plasma membrane actively participates in numerous cellular functions (Garcia-Parajo *et al*. 2014). Deciphering the dynamics of cell membranes on different time and spatial scales and knowing how much force is needed for cellular transport has become the key to understand biological functions of membranes (Lesniak *et al*. 2013). Owing to high signal-noise ratios, single molecule techniques have now become well-established tools for investigating the complex behaviors of different molecules on living cell membranes (Xie *et al*. 2006). These techniques can detect the structure, dynamics, and functions of single molecules, thus reveal their information that may be lost in ensemble averages. Here we discuss representative examples of using single molecule techniques to measure physicochemical parameters and uncover critical information on cell membranes (Ritort 2006). SMFS was used to detect the interaction force between the measured samples on the substrate and the molecules attached on the AFM tip, which happens during the AFM tip withdrawal from the sample surface.

An earlier pioneering work of single molecule techniques on the cell membrane is to image the epidermal growth factor receptor (EGFR) by single molecule fluorescence (Yasushi Sako 2000), which is lighted up by binding extracellular epidermal growth factor (EGF) labeled with Cy3. The investigators directly observed single molecular events in the plasma membrane by tracking each Cy3 spot. They found that the EGFR dimerization involved the formation of a complex composed of one EGF molecule and an EGFR dimer before the second EGF molecule binding. Single molecule fluorescence imaging was further used to study the binding kinetics of EGF to EGFR on living cell membranes (Teramura *et al*. 2006). A small number of EGFR on the cell surface formed dimeric binding sites, which interacted with EGF faster in two orders of magnitude than that of monomeric binding sites. The high association rate of the first binding event and the positive cooperativity of binding two EGFs to the predimeric sites facilitated the formation of signaling dimers. In the words, EGFR dimerization accelerates the binding between EGF and EGFR to active the downstream EGF signaling pathways. Also, a photobleaching-based method was developed to count single molecules in live cells (Ulbrich and Isacoff 2007). The number of fluorophores can be counted by observing the number of discrete photobleaching steps. This method provides a straight way to reveal the stoichiometry of membrane receptors. Because proteins are highly dynamic in living cells, single molecule fluorescence imaging of membrane proteins for their stoichiometric analysis often involves the use of single molecule tracking technique.

The single species of protein is free to diffuse only by the viscosity of the membrane, which is a key role in building stability and specific function of the cell membrane (Feder *et al*. 1996; Gudheti *et al*. 2013). To characterize the dynamics of specific molecules on the plasma membrane, a key parameter is the lateral diffusion coefficient (Shibata *et al*. 2006; Tani *et al*. 2005). Lino *et al.* proposed an oligomerization induced trapping model based on the results of quantized fluorescence intensity (Iino *et al*. 2001). Similar characterization was carried out for other proteins such as Ca^2+^ channel proteins (Harms *et al*. 2001), H-Ras (Lommerse *et al*. 2004), cyclic adenosine 3’,5’-monophosphate (cAMP) receptors (Ueda *et al*. 2001), and peptide-binding proteins (Vrljic *et* *al*. 2002).

In addition to the lateral diffusion coefficient, an interactive force is also an important parameter for the dynamics of specific molecules on the plasma membrane. AFM offers great potential for characterizing single molecules and presents advantages in measuring the interaction force of molecules (Muller and Dufrene 2008). Rankl *et al.* investigated the attachment of human rhinoviruses to the receptors of cell surface at the single molecule level by AFM (Rankl *et* *al*. 2008). Sequential binding of multiple receptors has also been verified from recordings of characteristic quantized force spectra, which have suggested that multiple receptors are bound to the virus in a timely manner. Subsequently, researcher integrated optical tweezers with AFM and molecular dynamics simulations to test a prevailing multivalent binding mode (Sauter *et al*. 1992) of influenza virus with cellular surface receptors (Sieben *et al*. 2012). The observed unbinding pathways were diverse and contain zipper-like as well as all-or-none unbinding events. Alsteens *et al.* showed an atomic force and confocal microscopy set-up that allows the surface receptor landscape of cells to be imaged, and the virus binding events within the first millisecond of contacting with the cell would be mapped at high resolution (<50 nm) (Alsteens *et al*. 2017b). They showed that the rabies virus multiple interacted with cell surface receptors and then presented a theoretical approach to contour the free-energy landscape of early binding events between virus and cell surface receptors. When the first bond is formed between the viral glycoprotein, its cognate cell surface receptor shows relatively low lifetime and free energy, but it increases as additional bonds form rapidly (≤1 ms) (Alsteens *et al*. 2017b). This multi-receptor bond quickly enhances the initial attachment of the virus to the host cell. Recently, the research group also observed similar receptor binding when studying the herpesvirus binding to cell surface receptors (Delguste *et al*. 2018).

The primary function of the cell membrane is to protect cells from their surroundings. All substances need across the cell membrane to enter the cells (Aderem and Underhill 1999; Doherty and McMahon 2009). Noticeably, the glucose molecule is transported into cells via its transporter (Pan *et al*. 2018). It takes time (10–40 ms) and force (~40 pN) to transport the single glucose molecule into cells via the glucose transporter. The endocytosis of polyamidoamine (PAMAM) nanoparticles used as a drug carrier was also studied. Yang *et al.* tracked their entry cell process, it was found that the duration of transporting the single PAMAM into living cells, ranges from 2.5 to 65 ms with the mean value of 19 ± 11 ms, and the corresponding force distributes in the range of 50–425 pN with the mean value of 172 ± 74 pN. The results mean that cellular uptake of the single PAMAM needs about 170 pN. The ~19 ms of duration and ~1.0 µm/s of speed unquestionably highlight the fact that the invagination of PAMAM nanoparticles is a quite rapid process (Yang *et al*. 2016; Zhou *et al*. 2018). Interestingly, the endocytosis process of inorganic nanoparticles (including gold nanoparticles (Ding *et al*. 2015), carbon quantum dots (Lu *et al*. 2019), and silicon quantum dots (Wang *et al*. 2019)) is similar to that of PAMAM nanoparticles. However, the force and duration are different for cells to uptake these particles due to the influences from the particle shape, size, surface charge, and the material properties (Niemeyer 2001; Peng *et al*. 2013; Tuerhong *et al*. 2017). Revealing the dynamic mechanism of trans-membrane transporting on living cells will provide more opportunities to diagnosis and therapy of membrane related diseases.

**Figure 5:**
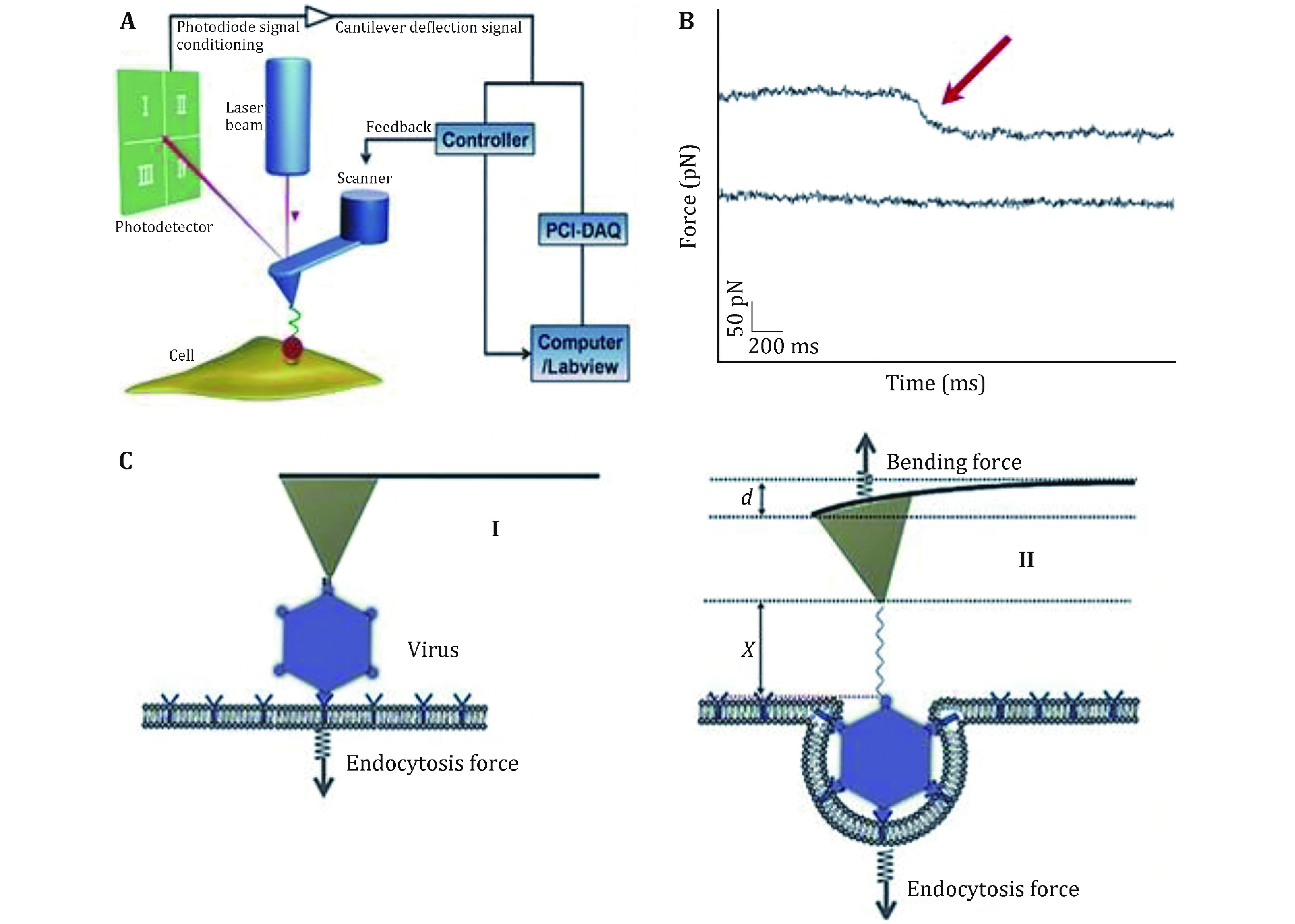
Schematic diagram of the force tracing technique for detecting the viral invagination. **A** Schematic diagram of the force tracing technique in constant position mode. The AFM tip modified with virions moves toward the cell surface and contacts the living cell at a constant position. **B** Typical force tracing curves (upper) of virus entry into the cell. No signals (lower) were observed when blocked with inhibitors. **C** The scheme of viral invagination via cell membranes. Reproduced from Pan *et al*. (2017) with permission of John Wiley & Sons Ltd

Most viruses need to be internalized into the cells through the endocytosis pathway to complete the infection after they attaching to the cell surface (Mercer *et al*. 2010). It is important to quantitatively characterize the underlying mechanism of this process. Pan *et al.* monitored the invasion process of single human enterovirus 71 by using the force tracing technique (Fig. 5A and 5B). They monitored the dynamic process of virus invagination, and found that it took about 280 ms for the invagination of single human enterovirus 71 virion. It is measured that the internalization force of single human enterovirus 71 is approximately 60 pN. The scheme of viral invagination via cell membranes is shown in Fig. 5C, where the displacement (*d* + *X*) was calculated (Pan *et al*. 2017). The force tracing technique was also used to monitor the dynamic process of single live Singapore grouper iridovirus (SGIV) invagination, and they found that it took about 1 s for the invagination of single SGIV with the maximum velocity of approximately 200 nm/s. It is measured that the internalization force of single SGIV is approximately 60 pN. In addition, the binding energy density becomes larger with the increasing of engulfment depth, indicating that additional binding events between viral ligands and receptors gradually occurred to provide enough energy for accomplishing virion invagination (Pan *et al*. 2015). The force tracing technique would provide a potential method to probe the entry cell dynamic mechanism of virus invasion.

## CONCLUSION AND OUTLOOK

Characterization of the multi-molecular structures of cell membranes is an essential step towards understanding its processes and functions, which could potentially lead to alternate applications in medicine and biotechnology (Escribá *et al*. 2008). Examples discussed here demonstrate that force and optical-based microscopy can tackle complex structures and functions with unprecedented resolution and sensitivity. Optical and force-based microscopies represent a powerful toolkit for studying the structure and functions of cell membranes at single molecule level.

Ultimately, the full potential of microscopies will be achieved when combining optical and force modalities. Establishing these correlated platforms in cell membranes should allow the identification and tracking of specific membrane components, while probing their biophysical properties (adhesion, transports, *etc.*) simultaneously on the same single cell, thus contributing to the important connection between their structure and functions (Wu *et al*. 2013b). Toward this goal, correlated AFM-fluorescence imaging technique has been exploited to track cell membrane dynamics at single molecule level (Cascione *et al*. 2017). For example, combing the force tracing with SIM, we can record the force tracing curves and locate the cell membrane receptors simultaneously during the trans-membrane transporting of targeted nano-drugs. These single molecule biophysics techniques based on optical and mechanical offer promising prospects for the comprehensive analysis of structures (including the distribution of membrane proteins and carbohydrates), dynamics (for example, nutrient endocytosis, virus intrusion, *etc*.), and interactions of single molecules (including ligand-receptor, antigen-antibody and so on) in the cell membrane. Meanwhile, helpful techniques, such as surface enhanced Raman spectroscopy and infrared spectroscopy should be combined with AFM to explore the more precise structure and functions of cell membranes (Bruzas *et al*. 2018; Mezzetti and Leibl 2017; Syed and Smith 2017). Furthermore, the cryo-electron microscopy with reconstituted three dimensional images also is hopeful to study the enzymatic molecular mechanisms and structure of single protein on complex cell membranes (Calder and Rosenthal 2016; Vinothkumar 2015; Zhao *et al*. 2020).

## Conflict of interest

Qingrong Zhang, Siying Li, Yu Yang, Yuping Shan and Hongda Wang declare that they have no conflict of interest.
